# A Case of Multiple Sclerosis and Celiac Disease

**DOI:** 10.1155/2013/576921

**Published:** 2013-01-13

**Authors:** H. Z. Batur-Caglayan, C. Irkec, I. Yildirim-Capraz, N. Atalay-Akyurek, S. Dumlu

**Affiliations:** ^1^Department of Neurology, Faculty of Medicine, Gazi Uniersity, 06500 Ankara, Turkey; ^2^Department of Pathology, Faculty of Medicine, Gazi Uniersity, 06500 Ankara, Turkey; ^3^Department of Gastroenterology, Faculty of Medicine, Gazi Uniersity, 06500 Ankara, Turkey

## Abstract

*Objectives*. Multiple sclerosis (MS) is an inflammatory autoimmune disorder of the central nervous system (CNS). Since a correlation between gluten intake and incidence of MS had been reported, the relationship of antigliadin antibodies and MS was debated. *Case Report*. We report the case of a 45-year-old female MS patient who is under interferon treatment. After seven years of monitoring, during her routine gastroenterological assessment, she was diagnosed with celiac disease. *Conclusion*. Beside the neurological manifestations that have been demonstrated in about 10% of celiac disease (CD) patients, white-matter abnormalities in brain MRI are uncommon and controversial. But in the literature, MS seems to be associated with CD as in our patient. We suggest that MS patients with gastroenterological complaints should undergo an assessment for CD.

## 1. Objectives

Multiple sclerosis (MS) is an inflammatory autoimmune disorder of the central nervous system (CNS). MS, resembling other autoimmune disorders, has a multifactorial etiology, including environmental, immunological, and genetic factors. MS is sometimes difficult to be differentiated from CNS involvement in systemic autoimmune diseases [1].

Celiac disease is an immune-mediated intestinal disorder with gluten sensitivity which is characterized with villous atrophy and crypt hyperplasia. Celiac disease is well known to be associated with many neurological diseases like cerebellar ataxia, peripheral neuropathy, epilepsy, dementia, and depression. Earlier reports mainly have documented the involvement of the nervous system as a complication of prediagnosed CD. As gluten sensitivity with or without intestinal involvement shows concurrence with neurological manifestations like white-matter lesions, MS has been studied for the association with gluten sensitivity [2–5]. We describe an MS patient who is diagnosed with CD after seven years of followup.

## 2. Case Report

**Figure 1:**
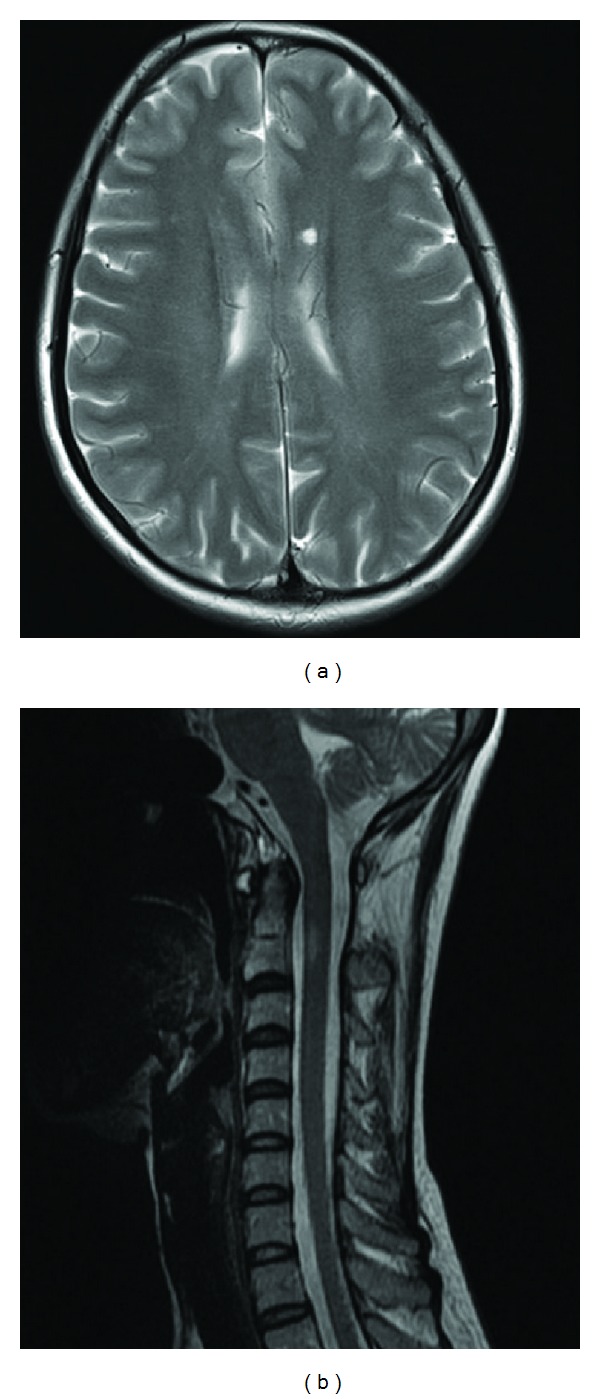
Brain MRI (a) and cervical spine MRI (b).

Seven years ago, at the age of 38, a female patient was consulted to our clinic with right leg weakness and paresthesias in her arms and legs. Neurological examination showed right hemiparesis (4/5) and right hemihypoesthesia. Past history was unremarkable except that she had irritable bowel syndrome and iron deficiency anaemia. Routine laboratory investigations revealed haemoglobin of 12,4 g/dL with MCV of 80,8 and serum ferritin 6,36 ng/mL (normal values 7–270 ng/mL) confirming a mild iron deficiency. Detailed biochemical and immunological profiles were normal. Vitamin B12, folate, ANA, anti-dsDNA antibodies, ANCA, ASMA, AMA, and anticardiolipin and antiphospholipid antibodies were normal. Brain MRI showed T1 isointense and T2 hyperintense abnormalities in deep periventricular white matter and left ventricular trigone (Figure 1(a)). Cervical spine MRI showed an expanding focal lesion at level C2 with gadolinium enhancement (Figure 1(b)). Cerebrospinal fluid investigation represents mildly increased IgG index but not with oligoclonal bands. Visual-, somatosensory-, and motor-evoked potentials assessments were normal. The patient was treated with intravenous methylprednisolone for 5 days. Motor and sensory symptoms gradually improved. After one year, she was readmitted to a local hospital in another city three times with symptoms like right hemiparesis, visual blurring in right eye, dizziness, and diplopia. She had received methyl prednisolone for ten days. Repeated brain MRI showed millimetric signal changes in periventricular region. According to Mc Donald's criteria, MS diagnosis had been made. Because of the relapsing course of disease, the presence of paresthesias, Uhthoff's phenomenon, and Lhermitte's sign, beta interferon therapy was planned. In a seven-year observation, two attacks as paraparesis and diplopia were seen. But the patient fully recovered after pulse steroid therapy. Neurological examination revealed no abnormality except right hemihypoesthesia and hyperactive deep tendon reflexes. But iron deficiency aenemia deteriorated. Then, haematology and gastroenterology consultations were repeated. Celiac screening for autoantibodies antigliadin antibody (AGA) IgA and tissue transglutaminase antibody (TTG) showed positivity. Gastrointestinal endoscopy revealed antral gastritis and duodenopathy. In biopsy material, blunting of villi and intraepithelial lymphocytes was consistent with celiac disease, MARSH type 3b. The patient was given gluten-free diet for celiac disease.

## 3. Conclusion

MS is known to be associated with other autoimmune diseases. Since gluten sensitivity and serum antigliadin (AGA), antiendomysium (AEA), and antitissue transglutaminase (AtTGA) antibodies had been studied in MS patients, most of the studies found the prevalence of antigliadin antibodies in MS patients was the same as in controls [1, 6–9]. On the other hand, CD presents neurological dysfunctions like ataxia, peripheral neuropathy, and epilepsy. Recently “multiple sclerosis-like diseases” and headache associated with “brain MRI white-matter lesions” have been reported in celiac patients [10]. Pengiran Tengah and friends describe two cases with MS-like disease with CD. They were treated with IV methyl prednisolone for 3 days, and they were commenced on gluten-free diet [11].

In our patient, intestinal symptoms were mild for years. Constipation and persistent iron deficiency did not suggest CD, and we did not perform any test for gluten sensitivity. Also, cranial and spinal lesions were not typical for MS. Multiple attacks in 1 year and no other clinical evidence for any other disease made us accept the diagnosis for MS. MRI did not show dissemination in time and space under interferon therapy.

MS and CD are considered T-cell-mediated autoimmune diseases, and the involvement of Th1 cells in their pathogenesis has been suggested. Frisullo et al. reported a patient with MS and CD, and they found the interaction between MS and CD related inflammatory processes may result in an amplfication of Th1 immune response [12]. However, in CD, activated HLA-restricted gliadin-specific T cells and antigliadin antibodies are found systemically. Antigliadin antibodies are also found in the CSF. They might be responsible for headache and white-matter abnormalities as cerebellar ataxia [10].

Hadjivassiliou et al. proposed that the MRI changes in their cases with MS-like disease were different than those seen in MS, being more peripherally situated and often confluent. They considered gluten sensitivity might be the etiology of “atypical” primary progressive MS particularly where ataxia is a prominent feature [13]. 

We describe our patient as an uncommon coincidence of MS and CD. Also, she can be diagnosed as having gluten sensitivity and MS-like disease associated with CD. We emphasize the importance of systemic examination and medical history. MS patients with gastroenterological complaints should be tested for gluten sensitivity. We suggest that the interferon therapy in MS-CD patients should also be considered with gluten-free diet.
